# Influenza and COVID-19 Vaccination Coverage Among Health Care Personnel — National Healthcare Safety Network, United States, 2023–24 Respiratory Virus Season

**DOI:** 10.15585/mmwr.mm7343a2

**Published:** 2024-10-31

**Authors:** Jeneita Bell, Lu Meng, Kira Barbre, Emily Wong, Brynn Lape-Newman, Wilson Koech, Minn M. Soe, Austin Woods, David T. Kuhar, Matthew J. Stuckey, Heather Dubendris, Theresa Rowe, Megan C. Lindley, Elizabeth J. Kalayil, Jonathan Edwards, Andrea Benin, Hannah E. Reses

**Affiliations:** ^1^Division of Healthcare Quality Promotion, National Center for Emerging and Zoonotic Infectious Diseases, CDC; ^2^Goldbelt C6, Chesapeake, Virginia; ^3^Lantana Consulting Group, East Thetford, Vermont; ^4^Chenega Enterprise Systems & Solutions, LLC, Chesapeake, Virginia; ^5^Immunization Services Division, National Center for Immunization and Respiratory Diseases, CDC.

**Summary**

**What is already known about this topic?**

The Advisory Committee on Immunization Practices (ACIP) recommends annual influenza vaccination for health care personnel. In September 2023, ACIP recommended receipt of a 2023–2024 COVID-19 vaccine for all persons aged ≥6 months.

**What is added by this report?**

During the 2023–24 respiratory virus season, influenza vaccination coverage was 80.7% among acute care hospital personnel and 45.4% among nursing home personnel. Coverage with 2023–2024 COVID-19 vaccination was 15.3% among acute care hospital personnel and 10.5% among nursing home personnel.

**What are the implications for public health practice?**

Respiratory viral diseases pose risks for health care personnel in U.S. health care settings, and vaccination is an effective strategy for maintaining a healthy workforce and improving health care system resiliency.

## Abstract

The Advisory Committee on Immunization Practices (ACIP) recommends that health care personnel receive an annual influenza vaccine. In September 2023, ACIP recommended that everyone aged ≥6 months receive a 2023–2024 COVID-19 vaccine. Health care facilities, including acute care hospitals and nursing homes, report vaccination of health care personnel against influenza and COVID-19 to CDC’s National Healthcare Safety Network (NHSN). During October 2023–March 2024, NHSN defined up-to-date COVID-19 vaccination as receipt of a 2023–2024 COVID-19 vaccine. This analysis describes influenza and 2023–2024 COVID-19 vaccination coverage among health care personnel working in acute care hospitals and nursing homes during the 2023–24 respiratory virus season (October 1, 2023–March 31, 2024). Influenza vaccination coverage was 80.7% among health care personnel at acute care hospitals and 45.4% among health care personnel at nursing homes. Coverage of 2023–2024 COVID-19 vaccination was 15.3% among health care personnel at acute care hospitals and 10.5% among health care personnel at nursing homes. Respiratory viral diseases including influenza and COVID-19 pose risks to health care personnel in U.S. health care settings, and vaccination of health care personnel is an effective strategy for maintaining a healthy workforce and improving health care system resiliency.

## Introduction

Health care personnel are at risk for work-related exposure to respiratory viral diseases, including influenza and COVID-19 (*1*). Vaccination of health care personnel helps maintain a healthy workforce (*2*) and reduces the risk for staffing shortages (*3*). The Advisory Committee on Immunization Practices (ACIP) recommends that health care personnel receive an annual influenza vaccine (*4*). In September 2023, ACIP recommended a 2023–2024 COVID-19 vaccine for all persons aged ≥6 months (*5*). The Centers for Medicare & Medicaid Services (CMS) monitors the implementation of these recommendations by requiring health care facilities, including nursing homes and acute care hospitals, to report influenza* and COVID-19^†^ vaccination coverage among health care personnel^§^ to CDC’s National Healthcare Safety Network (NHSN). This study examined influenza and 2023–2024 COVID-19 vaccination coverage among health care personnel working in acute care hospitals and nursing homes during the 2023–24 respiratory virus season.

## Methods

### Data Collection

Acute care hospitals and nursing homes report data to NHSN according to surveillance protocols for influenza and COVID-19 vaccination. Acute care hospitals and nursing homes began reporting COVID-19 vaccination among health care personnel in 2021. Acute care hospitals were required to report influenza vaccination among health care personnel beginning in 2013^¶^; skilled nursing facilities were required to report influenza vaccination among health care personnel beginning with the 2022–23 respiratory virus season. To determine influenza vaccination coverage, facilities report the total number of health care personnel working in the facility for ≥1 day during a respiratory virus season (October 1–March 31)** and the total number of health care personnel who 1) reported receipt of influenza vaccination, 2) had a medical contraindication to influenza vaccination, 3) declined vaccination, and 4) had unknown vaccination status. The protocol for COVID-19 vaccination coverage includes parallel data fields for COVID-19; however, data collection occurs at a different cadence. Nursing homes and acute care hospitals report on schedules mandated by their respective regulatory programs at CMS. Nursing homes submit COVID-19 vaccination coverage weekly^††^; acute care facilities submit ≥1 week of data per month.^§§^ Both types of facilities report COVID-19 vaccination coverage data among health care personnel who were eligible to work in the facility for ≥1 day during the reporting week. Because vaccination coverage data reported to NHSN are aggregated at the facility level, information on the percentage of health care personnel who were up to date with both influenza and 2023–2024 COVID-19 vaccination was not available.

### Data Analysis

To determine health care personnel vaccination coverage during the 2023–24 respiratory virus season, analyses were conducted using influenza and up-to-date COVID-19 vaccination coverage data (specifically, up-to-date COVID-19 vaccination coverage data from the week ending March 31, 2024, or the last submitted week of data) reported to NHSN from acute care hospitals and nursing homes in all 50 U.S. states. NHSN defined up-to-date COVID-19 vaccination as the receipt of ≥1 dose of a 2023–2024 COVID-19 vaccine.^¶¶ ^Facilities that reported data for both vaccine types were included in the analysis. Pooled mean vaccination coverage with influenza and COVID-19 was calculated as the number of health care personnel who reported receipt of each recommended vaccine divided by the number of health care personnel working in all facilities. Health care personnel reported to have a medical contraindication to receiving an influenza (0.89% of all health care personnel) or COVID-19 (0.71% of all health care personnel) vaccination were subtracted from the denominator of the vaccination coverage calculation for the corresponding vaccine. Coverage with each vaccine was calculated for health care personnel working at each facility type (nursing home and acute care hospital). Results were further stratified by employment category (employee, licensed independent practitioner, and student/trainee or volunteer); urban-rural classification (rural or urban)***; county-level social vulnerability index (SVI) tertile^†††^; facility size tertile^§§§^; state; and U.S. region.^¶¶¶^ Vaccination coverage for each vaccine type in each facility type was calculated for each U.S. state; results were categorized into levels based on the overall quintile distribution of coverage across both vaccines and both facility types. Counties in a lower SVI tertile are less socially vulnerable than are those in an upper SVI tertile. All analysis was conducted using SAS (version 9.4; SAS Institute). This activity was reviewed by CDC, deemed not research, and was conducted consistent with applicable federal law and CDC policy.****

## Results

### Influenza Vaccination Coverage: Acute Care Hospitals

Among approximately 8.8 million health care personnel working in 4,114 acute care hospitals, influenza vaccination coverage was 80.7% overall (Table 1); coverage was lowest (65.7%) among licensed independent practitioners. Vaccination coverage was highest in the Mountain region (84.5%) and lowest in the Pacific region (74.3%). Acute care hospitals in 38 states reported influenza vaccination coverage of ≥75% among health care personnel (Figure) (Supplementary Table; https://stacks.cdc.gov/view/cdc/166705).

**TABLE 1:** Pooled mean influenza vaccination coverage among health care personnel working at acute care hospitals and nursing homes, by facility type — National Healthcare Safety Network, United States, October 1, 2023–March 31, 2024*

Characteristic	Acute care hospitals				Nursing homes			
	No. of facilities	No. of HCP	No. of vaccinated HCP	Coverage % (95% CI)^†^	No. of facilities	No. of HCP	No. of vaccinated HCP	Coverage % (95% CI)^†^
**Total**	**4,114**	**8,827,687**	**7,124,797**	**80.7 (80.7–80.7)**	**14,294**	**2,141,284**	**971,963**	**45.4 (45.3–45.5)**
**Staff member type**								
Employee	4,113	6,560,940	5,487,078	83.6 (83.6–83.7)	14,294	1,942,270	863,430	44.5 (44.4–44.5)
Licensed independent practitioner	3,749	1,290,268	847,676	65.7 (65.6–65.8)	11,621	105,820	54,396	51.4 (51.1–51.7)
Student/trainee or volunteer	3,503	976,479	790,043	80.9 (80.8–81.0)	4,399	93,194	54,137	58.1 (57.8–58.4)
**Facility size^§^**								
Small	1,371	447,184	346,806	77.6 (77.4–77.7)	4,802	358,030	162,539	45.4 (45.2–45.6)
Medium	1,371	1,674,424	1,285,596	76.8 (76.7–76.8)	4,714	611,336	267,944	43.8 (43.7–44.0)
Large	1,372	6,706,079	5,492,395	81.9 (81.9–81.9)	4,778	1,171,918	541,480	46.2 (46.1–46.3)
**Urbanicity^¶^**								
Urban	2,917	8,000,649	6,466,545	80.8 (80.8–80.9)	10,376	1,698,189	779,250	45.9 (45.8–46.0)
Rural	1,197	827,038	658,252	79.6 (79.5–79.7)	3,918	443,095	192,713	43.5 (43.3–43.6)
Social vulnerability index**								
Low	1,242	2,557,867	2,112,743	82.6 (82.6–82.6)	4,893	703,749	336,549	47.8 (47.7–47.9)
Medium	1,359	3,223,133	2,572,998	79.8 (79.8–79.9)	4,776	761,881	337,303	44.3 (44.2–44.4)
High	1,512	3,046,079	2,438,500	80.1 (80.0–80.1)	4,623	675,527	298,002	44.1 (44.0–44.2)
**Region^††^**								
Midwest	1,053	2,134,165	1,781,857	83.5 (83.4–83.5)	4,613	612,962	241,100	39.3 (39.2–39.5)
Mountain	203	410,762	347,067	84.5 (84.4–84.6)	498	70,392	39,673	56.4 (56.0–56.7)
Northeast	580	1,697,515	1,412,808	83.2 (83.2–83.3)	2,348	464,122	272,044	58.6 (58.5–58.8)
Pacific	467	1,153,258	856,882	74.3 (74.2–74.4)	1,527	231,530	128,564	55.5 (55.3–55.7)
South	1,811	3,431,987	2,726,183	79.4 (79.4–79.5)	5,308	762,278	290,582	38.1 (38.0–38.2)

**Abbreviation:** HCP = health care personnel.

* Each facility reported summary influenza vaccination data among HCP working in the facility for ≥1 day during October 1, 2023–March 31, 2024. Coverage with 2023–2024 COVID-19 vaccination was reported to the National Healthcare Safety Network each week; data from the week ending March 31, 2024, or the last submitted week of data, were used for analysis.

^†^ 95% CIs were calculated using the mid-P method.

^§^ Facility size was calculated separately for acute care hospitals and nursing homes and was based on the tertile distribution of the total number of staff members per facility.

^¶^ https://www.cdc.gov/nchs/data-analysis-tools/urban-rural.html

** https://www.atsdr.cdc.gov/placeandhealth/svi/index.html

^††^ *Midwest*: Illinois, Indiana, Iowa, Kansas, Michigan, Minnesota, Missouri, Nebraska, North Dakota, Ohio, South Dakota, and Wisconsin; *Mountain*: Colorado, Idaho, Montana, Nevada, Utah, and Wyoming; *Northeast*: Connecticut, Maine, Massachusetts, New Hampshire, New Jersey, New York, Pennsylvania, Rhode Island, and Vermont; *Pacific*: Alaska, California, Hawaii, Oregon, and Washington; *South*: Alabama, Arizona, Arkansas, Delaware, Florida, Georgia, Kentucky, Louisiana, Maryland, Mississippi, New Mexico, North Carolina, Oklahoma, South Carolina, Tennessee, Texas, Virginia, and West Virginia.

**FIGURE:**
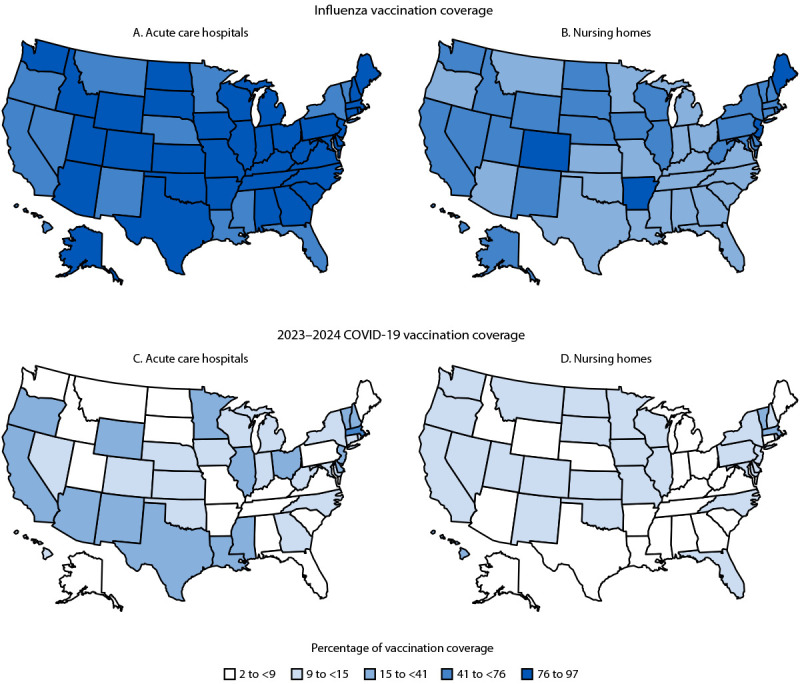
Percentage of pooled mean influenza vaccination coverage and 2023–2024 COVID-19 vaccination coverage among health care personnel working at acute care hospitals (A and C) and nursing homes (B and D), by facility type and state — National Healthcare Safety Network, United States, October 1, 2023–March 31, 2024* * Each facility reported summary influenza vaccination data among health care personnel working in the facility for ≥1 day during October 1, 2023–March 31, 2024. Coverage with 2023–2024 COVID-19 vaccination was reported to the National Healthcare Safety Network each week; data from the week ending on March 31, 2024, or the last submitted week of data, were used for analysis. State-level vaccination coverage results were categorized into levels based on the overall quintile distribution of coverage across both vaccines and both facility types.

### Influenza Vaccination Coverage: Nursing Homes

Among approximately 2.1 million health care personnel working in 14,294 nursing homes, influenza vaccination coverage was 45.4% overall; coverage was highest among students/trainees and volunteers (58.1%) and was lowest among employees (44.5%) (Table 1). Vaccination coverage was highest in the Northeast region (58.6%) and lowest in the South region (38.1%). Nursing homes in five states reported influenza vaccination coverage of ≥75% among health care personnel (Figure) (Supplementary Table; https://stacks.cdc.gov/view/cdc/166705).

### Coverage with COVID-19 Vaccination: Acute Care Hospitals

Among approximately 8.0 million health care personnel working in 4,112 acute care hospitals, 2023–2024 COVID-19 vaccination coverage was 15.3% overall (Table 2); coverage was lowest (12.7%) among licensed independent practitioners. Vaccination coverage was highest in large-sized facilities (16.1%) and in urban (15.6%) and high SVI (19.9%) areas. Coverage was highest in the Pacific region (20.9%) and lowest in the Mountain region (9.3%). Acute care hospitals in 12 states reported 2023–2024 COVID-19 vaccination coverage of ≥20% among health care personnel (Figure) (Supplementary Table; https://stacks.cdc.gov/view/cdc/166705).

**TABLE 2:** Pooled mean 2023–2024 COVID-19 vaccination coverage among health care personnel working at acute care hospitals and nursing homes, by facility type — National Healthcare Safety Network, United States, October 1, 2023–March 31, 2024*

Characteristic	Acute care hospitals				Nursing homes			
	No. of facilities	No. of HCP	No. of vaccinated HCP	Coverage % (95% CI)^†^	No. of facilities	No. of HCP	No. of vaccinated HCP	Coverage % (95% CI)^†^
**Total**	**4,112**	**7,958,264**	**1,215,283**	**15.3 (15.2–15.3)**	**14,281**	**1,783,878**	**187,529**	**10.5 (10.5–10.6)**
**Staff member type**								
Employee	4,107	6,084,708	966,371	15.9 (15.9–15.9)	14,278	1,663,089	165,774	10.0 (9.9–10.0)
Licensed independent practitioner	3,490	1,256,858	159,264	12.7 (12.6–12.7)	10,334	84,276	15,875	18.8 (18.6–19.1)
Student/trainee or volunteer	3,131	616,698	89,648	14.5 (14.4–14.6)	3,340	36,513	5,880	16.1 (15.7–16.5)
**Facility size^§^**								
Small	1,370	435,181	66,880	15.4 (15.3–15.5)	4,798	339,502	37,839	11.1 (11.0–11.3)
Medium	1,371	1,587,262	192,435	12.1 (12.1–12.2)	4,708	527,747	59,966	11.4 (11.3–11.4)
Large	1,371	5,935,821	955,968	16.1 (16.1–16.1)	4,775	916,629	89,724	9.8 (9.7–9.8)
**Urbanicity^¶^**								
Urban	2,916	7,203,900	1,124,506	15.6 (15.6–15.6)	10,366	1,416,806	158,896	11.2 (11.2–11.3)
Rural	1,196	754,364	90,777	12.0 (12.0–12.1)	3,915	367,072	28,633	7.8 (7.7–7.9)
Social vulnerability index**								
Low	1,242	2,296,349	315,278	13.7 (13.7–13.8)	4,887	588,459	66,178	11.2 (11.2–11.3)
Medium	1,358	2,912,226	352,632	12.1 (12.1–12.1)	4,770	635,212	63,125	9.9 (9.9–10.0)
High	1,511	2,749,110	547,184	19.9 (19.9–20.0)	4,622	560,081	58,217	10.4 (10.3–10.5)
**Region^††^**								
Midwest	1,053	1,865,337	307,453	16.5 (16.4–16.5)	4,609	507,102	48,856	9.6 (9.6–9.7)
Mountain	203	367,061	33,971	9.3 (9.2–9.3)	498	53,921	6,811	12.6 (12.4–12.9)
Northeast	580	1,596,904	284,339	17.8 (17.7–17.9)	2,348	405,581	50,696	12.5 (12.4–12.6)
Pacific	467	1,083,448	226,271	20.9 (20.8–21.0)	1,527	194,093	27,592	14.2 (14.1–14.4)
South	1,809	3,045,514	363,249	11.9 (11.9–12.0)	5,299	623,181	53,574	8.6 (8.5–8.7)

**Abbreviation:** HCP = health care personnel.

* Each facility reported summary influenza vaccination data among HCP working in the facility for ≥1 day during October 1, 2023–March 31, 2024. Coverage with 2023–2024 COVID-19 vaccination was reported to the National Healthcare Safety Network each week; data from the week ending March 31, 2024, or the last submitted week of data, were used for analysis.

^†^ 95% CIs were calculated using the mid-P method.

^§^ Facility size was calculated separately for acute care hospitals and nursing homes and was based on the tertile distribution of the total number of staff members per facility.

^¶^ https://www.cdc.gov/nchs/data-analysis-tools/urban-rural.html

** https://www.atsdr.cdc.gov/placeandhealth/svi/index.html

^††^ *Midwest*: Illinois, Indiana, Iowa, Kansas, Michigan, Minnesota, Missouri, Nebraska, North Dakota, Ohio, South Dakota, and Wisconsin; *Mountain*: Colorado, Idaho, Montana, Nevada, Utah, and Wyoming; *Northeast*: Connecticut, Maine, Massachusetts, New Hampshire, New Jersey, New York, Pennsylvania, Rhode Island, and Vermont;* Pacific*: Alaska, California, Hawaii, Oregon, and Washington; *South*: Alabama, Arizona, Arkansas, Delaware, Florida, Georgia, Kentucky, Louisiana, Maryland, Mississippi, New Mexico, North Carolina, Oklahoma, South Carolina, Tennessee, Texas, Virginia, and West Virginia.

### Coverage with 2023–2024 COVID-19 Vaccination: Nursing Homes

Among approximately 1.8 million health care personnel working in 14,281 nursing homes, 2023–2024 COVID-19 vaccination coverage was 10.5% overall (Table 2); coverage was highest among licensed independent practitioners (18.8%) and lowest among employees (10.0%). Vaccination coverage was highest among those working in the Pacific region (14.2%) and lowest among those working in the South region (8.6%). Nursing homes in two states reported vaccination coverage of ≥20% among health care personnel (Figure) (Supplementary Table; https://stacks.cdc.gov/view/cdc/166705).

## Discussion

During the 2023–24 respiratory virus season, fewer than one in six health care personnel working in acute care hospitals and nursing homes reported receipt of a 2023–2024 COVID-19 vaccine, and fewer than one half of health care personnel working in nursing homes had received an influenza vaccine. Coverage with influenza and 2023–2024 COVID-19 vaccination was higher among health care personnel in acute care hospitals than among those in nursing homes. Although characteristics associated with vaccination varied by vaccine and facility type, coverage was generally higher in urban than in rural areas and lower in the South.

Coverage with COVID-19 vaccination among health care personnel in nursing homes decreased from 22.8% during the 2022–23 respiratory virus season to 10.5% during the 2023–24 respiratory virus season (*6*). During the same period, COVID-19 vaccination coverage among health care personnel in acute care hospitals decreased from 17.8% to 15.3%. Compared with vaccination during the 2022–23 respiratory virus season, influenza vaccination among health care personnel in acute care hospitals remained stable at approximately 81%; this percentage remains well below the 91% coverage reported for the 2019–20 season, indicating that influenza vaccination coverage among health care providers remains persistently below the levels during the prepandemic period (*7*).

This study identified a marked decrease in COVID-19 vaccination coverage among health care personnel in nursing homes from the 2022–23 respiratory virus season to the 2023–24 respiratory virus season. CMS’s regulatory requirement for vaccination of health care personnel against COVID-19 expired in June 2023^††††^ and, in fall 2023, COVID-19 vaccines were commercialized.^§§§§^ These two events might have affected vaccination campaigns and on-site access to COVID-19 vaccines in nursing homes, and commercialization of the vaccine increased costs for facilities and health care personnel. In addition, a recent survey of health care personnel indicated that, although personnel believe that COVID-19 is a serious health threat, they have low confidence in the effectiveness, safety, and benefit of COVID-19 vaccination (*8*). In one study, health care personnel who felt sufficiently informed about the COVID-19 vaccine were 10 times more likely to receive COVID-19 vaccination and four times more likely to recommend the vaccine to their patients (*9*). This finding suggests that if vaccines are readily available, education might play an important role in improving vaccine confidence and vaccination coverage.

Among health care personnel working in acute care hospitals, coverage with both COVID-19 and influenza vaccination was lowest among licensed independent practitioners. This finding underscores ongoing challenges with vaccination of nonemployee health care personnel and documentation of vaccination that occurs outside of the facility (*10*). Like findings in previous studies, the current findings highlight the need to further investigate barriers to vaccination among health care personnel and identify additional strategies to address these challenges. For example, a recent study found that multifaceted campaigns that include on-site vaccination are effective at increasing vaccination coverage among health care personnel (*10*).

### Limitations

The findings in this report are subject to at least four limitations. First, influenza and COVID-19 vaccination coverage were reported separately using different definitions of total health care personnel working in the facility. The proportion of health care personnel who were included in the coverage calculations for both seasonal influenza vaccination coverage and weekly COVID-19 vaccination coverage is unclear. This lack of clarity limits the direct comparability of coverage with the two vaccines; therefore, statistical comparisons between influenza and COVID-19 vaccination coverage were not conducted. Second, this report includes data reported by facilities on behalf of health care personnel, which might have resulted in underestimates of vaccination acquired outside the health care facility. Third, vaccination coverage could not be stratified by recent history of SARS-CoV-2 infection. CDC recommendations state that persons might consider delaying an updated vaccine by 3 months after experiencing SARS-CoV-2 infection.^¶¶¶¶^ Therefore, some personnel might have declined vaccination against COVID-19 after a recent infection with SARS-CoV-2. Finally, this analysis was conducted using aggregate data reported to NHSN at the facility level; therefore, vaccination coverage could not be stratified by person-level covariates that might potentially enable an assessment of differences in coverage by factors such as age, race and ethnicity, or job category.

### Implications for Public Health Practice

Although the COVID-19 public health emergency has ended, thousands of COVID-19–related hospitalizations and hundreds of COVID-19–associated deaths still occur weekly.***** Influenza vaccination among health care personnel has not returned to 2019 levels, and the number of COVID-19 vaccinations has continued to decline each season, underscoring the ongoing challenge of promoting vaccination among health care personnel during the postpandemic period. Studies are needed to identify effective strategies to improve vaccination at a time when health care personnel are susceptible to low vaccine confidence. Also, improving confidence about the safety and effectiveness of vaccines among health care personnel through, for example, providing additional education about the safety and effectiveness of vaccination to health care personnel (*9*), has various benefits beyond increased vaccination coverage, such as decreased risk for staffing shortages (*3*) and increased patient vaccination (*9*). Respiratory viral diseases including influenza and COVID-19 pose risks to health care personnel in U.S. health care settings, and vaccination of health care personnel is an effective strategy for maintaining a healthy workforce (*1*,*2*) and improving health care system resiliency (*3*).

## Footnotes

*
https://www.cdc.gov/nhsn/faqs/vaccination/faq-influenza-vaccination-summary-reporting.html

†
https://www.cdc.gov/nhsn/pdfs/hps/covidvax/2024-hcp-combined-protocol-508.pdf

§
https://www.cdc.gov/nhsn/pdfs/covid19/covidvax-staff-toi-508.pdf

¶
https://www.cdc.gov/nhsn/pdfs/cms/vaccination/operational-guidance-ach-hcp-flu-508.pdf

**
https://www.govinfo.gov/content/pkg/FR-2011-08-18/pdf/2011-19719.pdf

††
https://www.federalregister.gov/documents/2021/05/13/2021-10122/medicare-and-medicaid-programs-covid-19-vaccine-requirements-for-long-term-care-ltc-facilities-and

§§
https://www.federalregister.gov/documents/2021/08/13/2021-16519/medicare-program-hospital-inpatient-prospective-payment-systems-for-acute-care-hospitals-and-the?form=MY01SV&OCID=MY01SV

¶¶
https://www.cdc.gov/nhsn/pdfs/hps/covidvax/UpToDateGuidance-508.pdf

***
https://www.cdc.gov/nchs/data-analysis-tools/urban-rural.html

†††
https://www.atsdr.cdc.gov/placeandhealth/svi/index.html

§§§ Facility-size tertile was calculated separately for acute care hospitals and nursing homes and was based on the distribution of the total number of staff members per facility.

¶¶¶ *Midwest*: Illinois, Indiana, Iowa, Kansas, Michigan, Minnesota, Missouri, Nebraska, North Dakota, Ohio, South Dakota, and Wisconsin; *Mountain*: Colorado, Idaho, Montana, Nevada, Utah, and Wyoming; *Northeast*: Connecticut, Maine, Massachusetts, New Hampshire, New Jersey, New York, Pennsylvania, Rhode Island, and Vermont; *Pacific*: Alaska, California, Hawaii, Oregon, and Washington; *South*: Alabama, Arizona, Arkansas, Delaware, Florida, Georgia, Kentucky, Louisiana, Maryland, Mississippi, New Mexico, North Carolina, Oklahoma, South Carolina, Tennessee, Texas, Virginia, and West Virginia.

**** 45 C.F.R. part 46, 21 C.F.R. part 56; 42 U.S.C. Sect. 241(d); 5 U.S.C. Sect. 552a; 44 U.S.C. Sect. 3501 et seq.

††††
https://www.cms.gov/files/document/qso-23-02-all-expired.pdf

§§§§
https://www.hhs.gov/coronavirus/commercialization/index.html

¶¶¶¶
https://www.cdc.gov/covid/vaccines/stay-up-to-date.html

***** https://covid.cdc.gov/covid-data-tracker/?os = vb.&ref = app#datatracker-home (Accessed October 29, 2024).
